# Disseminated fusariosis with ecthyma gangrenosum-like lesions in a refractory acute myeloid leukemia patient

**DOI:** 10.18502/cmm.5.1.534

**Published:** 2019-03

**Authors:** Masoud Mardani, Rozita Khodashahi, Ensieh Lotfali, Sara Abolghasemi, Mojhde Hakemi-vala

**Affiliations:** 1 Infectious Diseases and Tropical Medicine Research Center, Shahid Beheshti University of Medical Sciences, Tehran, Iran; 2 Department of Medical Parasitology and Mycology, School of Medicine, Shahid Beheshti University of Medical Sciences, Tehran, Iran; 3 Department of Microbiology, School of Medicine, Shahid Beheshti University of Medical Sciences, Tehran, Iran

**Keywords:** Disseminated fusariosis, Ecthyma gangrenosum-like lesions, Fungemia

## Abstract

**Background and Purpose:**

*Fusarium *species is an opportunistic mold that causes disseminated infections in immunocompromised patients. Given the high mortality rate of this infection, it is important to make a definite diagnosis when encountering suspected cases.

**Case report:**

Herein, we presented a 35-year-old man diagnosed with acute myeloid leukemia with a prolonged febrile neutropenic period and ecthyma gangrenosum-like lesions, along with fungemia and disseminated fusariosis. The patient died despite receiving combination therapy, perhaps due to the nonrecovery of neutrophil.

**Conclusion:**

Ecthyma gangrenosum-like lesions due to disseminated fusariosis might be easily misdiagnosed. Consequently, more attention should be paid to the cutaneous lesions in immunocompromised patients.

## Introduction

*Fusarium *species is an opportunistic mold that causes a wide range of infections. In immunocompromised patients, disseminated fusariosis is a serious infection resulting in a high mortality rate [1, 2]. Cutaneous lesions, such as ecthyma gangrenosum, are seen in 70% of the cases inflicted with this infection [1]. Histopathological and microbiological features of the skin lesions have a critical role in differential diagnosis [2, 3]. Herein, we presented a patient with acute myeloid leukemia (AML) and ecthyma gangrenosum-like cutaneous lesions who simultaneously developed fungemia and disseminated fusariosis. The present study aimed to address the cutaneous lesions in immunocompromised patients as a cause of diagnostic challenge and their association with different invasive etiologic agents while having similar appearances.

## Case report

A 35-year-old man with refractory AML was admitted to a tertiary hospital in Tehran, Iran. He was a candidate for EMA regimen, including mitoxantrone, etoposide, and cytarabine. The patient was subjected to central venous catheter and chemotherapy. Four days after chemotherapy, he became feverish due to catheter-related infection with an oral temperature of 38.3°C and an absolute neutrophil count of less than 100 cell/μl. 

Laboratory evaluation also revealed anemia and thrombocytopenia with a hemoglobin level of 9.5 g/dl and platelet count of 20,000 per microliter. The results of urinalysis were normal, and meropenem and teicoplanin were prescribed. Ultrasonography revealed acute thrombosis in the jugular vein. Furthermore, *Staphylococcus epidermidis *was detected in the blood cultures of the central line and peripheral vein*.* Antibiotic lock therapy was started simultaneously with systemic antibiotics. The patient became afebrile after 3 days.

**Figure 1:**
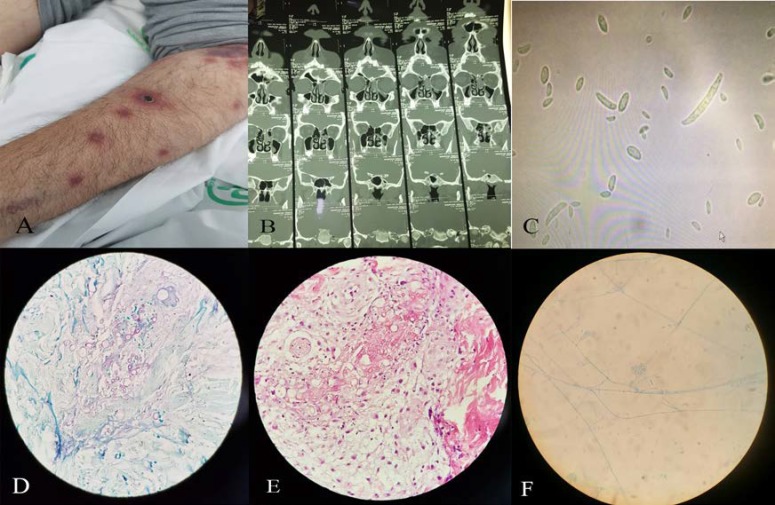
Lesions with a necrotic center resembling ecthyma gangrenosum on the upper limbs, a) paranasal sinus computed tomography scan showing the opacification of the ethmoid and maxillary sinuses, b) lactophenol cotton blue staining of the blood isolate, c) classical ''banana-shaped'' macroconidia of Fusarium species, d and e) culture of skin biopsy with H/E and blue methylene stain, and f) culture of sinus biopsy

Ten days later, the patient had another episode of fever and neutropenia. However, other vital signs were stable. Paranasal sinuses computed tomography (CT) scan showed sinusitis generally at the maxillary and ethmoid sinuses with hyperdense opacification (Figure 1B). Liposomal amphotericin B (LAMB) with a dosage of 5 mg/kg was initiated, and he was subjected to sinus endoscopy and functional endoscopic sinus surgery. 

Simultaneously, multiple painful erythematous macular and papular lesions with a necrotic center resembling ecthyma gangrenosum were detected on the lower extremities, upper limbs, and trunk, which were then distributed to the head and neck (Figure 1A). Therefore, he was subjected to skin lesion biopsy. Furthermore, the patient complained of the loss of vision in his left eye, and fundoscopic examination revealed endophthalmitis. As a result, intravitreal AMB was added to the systemic antifungal therapy. 

While fever and neutropenia were still persisting, 10 ml of venous blood sample was aseptically obtained from the patient via venipuncture according to a standard technique by using a sterile syringe after skin disinfection. Subsequently, the inoculated culture bottles were placed into the BacT/ALERT Microbial Detection System (BacT/ALERT FA Plus, bioMerieux SA, France).

The BACTEC bottle that showed a sign of fungal growth was subcultured on the plates with the brain heart infusion agar (Merck, Germany) and Sabouraud dextrose agar (SDA) (Merck, Germany) separately, and then incubated at 37C and 30C sequentially. After 72 h, the results of blood culture on the brain heart infusion agar demonstrated a colony of fungi, microscopically seen in methylene blue (Figure 1C). The results of culture on SDA media showed the production of hyaline, banana-shaped multicellular macroconidia with foot cell at the base. 

DNA was extracted from the fresh and pure culture colonies using the method described by Makimura et al. with some modifications [4]. Briefly, small amount (approximately 5 mm^3^) of the fresh colony was allocated in 100 μL lysis buffer (100 mM Tris-HCl, pH=7.5, 30 mM EDTA, 0.5% w/v SDS) and crushed with a conical grinder (Micro Multi Mixer, IEDA Co. Ltd., Tokyo, Japan) for 1 min. Subsequently, it was incubated for 15 min at 100°C, mixed with 100 μL of 2.5 M sodium acetate, kept at -20°C for 60 min, and finally centrifuged at 12,000 g for 5 min. 

After the removal of supernatants, DNA was precipitated with an equal volume of isopropanol, and then kept at -20°C for 30 min and centrifuged at 8,000 g for 15 min. Then, the pellet was washed with 300 μL of 100% and 70% ethanol and air dried. Finally, the DNA was resuspended in 50 μL of ultrapure water and kept at -20°C until being used as template for polymerase chain reaction (PCR). The ITS1 and ITS4 universal primers were used for PCR.

In the next stage, DNA sequencing was performed for the accurate identification of the isolate (Bioneer Company, South Korea). For the confirmation of species identity, the obtained sequences were compared with similar sequences in the open access NCBI database (http://blast.ncbi.nlm.nih.gov/Blast.cgi). Alignment of the obtained sequence in BLAST revealed a 99% identicality with the type strain of *Fusarium chlamydosporum*, indicated with sequence ID: KX783374.1. 

The sequences were in GenBank under the accession number ‘‘MK212931.’’ Microdilution testing was performed according to the CLSI document M38-A2 [5]. The antifungal agents administered were AMB (Sigma-Aldrich, USA), voriconazole (VOR; Pfizer Central Research, UK), and caspofungin (Merck, USA). The endpoint was the antifungal concentration that produced complete inhibition of visual growth during 48 h. The minimum inhibitory concentration (MIC) endpoint for the VOR and AMB was defined as the lowest concentration that produced complete growth inhibition. 

On the other hand, the minimum effective concentration (MEC) endpoint for caspofungin was defined based on the previous research [5, 6]. The MEC is the lowest concentration of drug that leads to the growth of small, rounded, compact hyphal forms as compared to the hyphal growth seen in the growth control well.* Fusarium *species was susceptible to AMB, VOR, and caspofungin.

After the removal of the catheter, VOR 6 mg/kg (twice a day) was administered for the first day, and then 4 mg/kg (twice a day) was prescribed, in addition to LAMB. Serum galactomannan test results were found to be positive twice consecutively. Histopathological analysis of the skin lesion on the lower extremity revealed deep-sited supportive granulomatous inflammatory dermal reaction, containing mycelial fungal element, which was consistent with deep mycosis. 

The smear revealed hyphae that were compatible with *Fusarium *species (Figure 1D and E). Sinus biopsy revealed sinonasal mucosa with fragmental elements compatible with mold infection. Furthermore, the sinus biopsy culture demonstrated hyaline septate hyphae, which were suspected as *Fusarium *species according to the phenotypic criteria (i.e., microscopic and macroscopic characteristics; Figure 1F). Eight days after the initiation of antifungal combination therapy, the patient’s general condition worsened, and he developed respiratory distress. Therefore, the patient was transferred to the Intensive Care Unit; however, he passed away.

## Discussion

*Fusarium *species are common agents of onychomycosis and fungal keratitis, which can cause severe disease, particularly in immunocompromised patients [1-3]. Immunocompromised patients with such conditions as organ transplantation, hematological malignancy, and prolonged neutropenia, are at a greater risk of invasive fusariosis. Furthermore, these patients typically have a poor prognosis with mortality rates exceeding 50% [7-9]. Disseminated fusariosis is described as the involvement of two or more noncontiguous regions [10]. 

Skin involvement is the first clue in most of the disseminated fusariosis cases and often occurs at an early stage of the disease [9]. Multiple painful erythematous macular or papular lesions are reported in 70% of the cases [1]. Lesions usually have a necrotic center resembling ecthyma gangrenosum and are described as ecthyma gangrenosum-like lesions [1]. In immunocompromised patients*, *fusariosis often disseminates and commonly involves the lungs [1, 11]. 

It is crucial to differentiate between *Fusarium* and *Aspergillus* species due to differences in their therapeutic strategies and prognoses. Negativity of galactomannan antigen and absence of the typical radiological signs of *Aspergillosis* can be in favor of fusariosis diagnosis. Recent studies have reported that galactomannan can be positive in cases with fusariosis [11, 12].

Sinusitis, a common manifestation of invasive fusariosis in hematologic malignancy patients, can be also diagnosed with CT imaging [13]. Fusariosis sinusitis generally involves the maxillary and ethmoid sinuses. Disseminated fusariosis, unlike disseminated *Aspergillosis* or zygomycetes infection, can be diagnosed through blood culture [1, 3, 14]. The propensity of *Fusarium *species to disseminate is assumed to be due to the yeast-like forms produced by the fungus that allows breakthrough into the bloodstream and subsequent growth [14].

Molecular assays, including PCR (generally followed by sequencing) and antigen detection assays (cell wall components), have been developed for the identification of *Fusarium *species at the species level [15, 16]. Most of the invasive fusariosis cases are caused by *Fusarium solani*, *Fusarium oxysporum*, and *Fusarium moniliforme*. To the best of our knowledge, two cases of human infection by *Fusarium chlamydosporum* have been reported so far [17, 18]. One of them was a catheter-associated infection in a patient with lymphoma [17] and another one was invasive sinusitis in an aplastic anemia patient [18]. 

The empiric therapy for invasive fusariosis infections includes either VOR or LAMB, surgical debridement (if possible), and posaconazole (POS) for salvage therapy [1, 19]. The LAMB may be used singly; however, successful results have been also achieved by combining it with VOR , POS, terbinafine, or natamycin [9, 20-23]. Luliconazole and lanoconazole are novel topical FDA-approved imidazoles for the treatment of superficial mycoses. 

These drugs have been reported to show in vitro activities against most of the molds and yeasts. In a study, luliconazole (<0.125 g/ml) and lanoconazole (<1 g/ml) showed low MIC values for all clinical *Fusarium *species [24]. For our case, VOR was prescribed to be used in addition to LAMB.

Treatment of disseminated fusariosis can be difficult since the genus is often multidrug-resistant [2, 25, 26]. Accordingly, a successful therapy of this disease requires immune reconstitution [1, 26]. In our case, cutaneous involvement, endophtalmitis, fungemia, and sinusitis were consistent with disseminated fusariosis and associated with the nonimprovement of neutrophil count, resulting in poor outcomes.

## Conclusion

It is crucial to carefully detect every single skin lesion in patients with hematological malignancies. Disseminated fusariosis would easily be overlooked unless implementing tissue cultures and histopathologic examination on the affected site.
